# Comparison of the Effect of Different Local Analgesia Administration Methods in Percutaneous Vertebroplasty: A Retrospective Cohort Study

**DOI:** 10.3389/fsurg.2022.769102

**Published:** 2022-03-25

**Authors:** Jiangxia Xiang, Weiyang Zhong, Yunsheng Ou

**Affiliations:** ^1^Department of Orthopedic Surgery, The First Affiliated Hospital of Chongqing Medical University, Chongqing, China; ^2^Department of Traumatology, Chongqing Emergency Medical Center, Chongqing University Central Hospital, Chongqing, China

**Keywords:** osteoporotic vertebral compression fractures, percutaneous vertebroplasty, local anesthesia, pain, safety

## Abstract

**Objective:**

Although various studies have described the methods of administering anesthesia during percutaneous vertebroplasty (PV) for treating osteoporotic vertebral compression fractures (OVCFs), there is still no consensus on the optimal treatment regimen. Therefore, this study aimed to investigate the effects of three application methods of local analgesia administration in PV for treating OVCFs.

**Methods:**

A total of 96 patients with OVCFs were reviewed and divided into three groups (A: lidocaine, B: ropivacaine, C: lidocaine + ropivacaine). The visual analog scale (VAS), blood pressure (BP), heart rate (HR), blood oxygen saturation (BOS), and surgery time were recorded during the following different points: before puncture, during the puncture, cement injection, and 4-h after surgery.

**Results:**

The mean age of the patients was 74.13 ± 7.02 years in group A, 70.47 ± 5.50 years in group B, and 73.07 ± 7.51 years in group C, without significant difference. No significant differences were found in sex, age, hospital stay, surgery time, blood loss, and cement volume of the patients. In the periods of before puncture and 4-h after surgery, the VAS in group C decreased significantly than that in the periods of the puncture, cement injection, and immediately after surgery. Overall, there were no significant differences in systolic BP, diastolic BP, HR, and BOS during the different periods among the groups except HR in the period of the puncture in group C, which was slower than that in other groups, and HR in the period of cement injection in group A, which was faster than the other two groups. A correlation was observed between the VAS and the periods of cement injection (*r* = 0.5358) and after surgery (*r* = 0.5775) in group C.

**Conclusion:**

Compared with the other two methods, the use of lidocaine in combination with ropivacaine could effectively relieve intraoperative pain, making the patients feel more comfortable and experience better recovery.

## Introduction

Osteoporotic vertebral compression fractures (OVCFs) are a common type of fracture event with gradually increasing rates of morbidity and mortality in older adults. A total of 1.4 million new fractures are estimated to occur every year worldwide (1, 2). OVCFs have been treated with conservative management, such as bed rest, analgesics, and braces, and with one of the following surgical procedures, percutaneous vertebroplasty (PV) or percutaneous kyphoplasty (PK) or segmental instrumentation. Besides, PV is usually performed under general anesthesia or local anesthesia (3–5). However, more and more older patients with underlying diseases suffer from OVCFS, and the patients are facing a higher risk when under general anesthesia. Local anesthesia not only facilitates the communication and cooperation between the surgeons and patients during the operation, and the surgeons can observe the surgery safety, but also reduces the surgery risks. However, local anesthesia still faces problems such as poor effect and poor patient experience. How to improve the effectiveness of local anesthesia still remains an issue (6–10). In our study, we aimed to investigate the clinical importance and the effect of the three different methods of local anesthetic drugs administration in PV treating the OVCFs.

## Methods

This study was approved by the Institutional Review Board of The First Affiliated Hospital of Chongqing Medical University and conducted according to the principles of the Declaration of Helsinki. All the patients provided their written informed consent to participate and were divided into three groups in our study prior to the storage of their data in the hospital database. From January 2019 to June 2019, 96 patients with OVCFs were treated in our department. All surgical procedures were performed by the same senior surgeon. In group A, 33 patients received PV using local anesthesia of the lidocaine; in group B, 31 patients received the local anesthesia of the ropivacaine; and in group C, 32 patients received the local anesthesia of the lidocaine in combination with the ropivacaine. The inclusion criteria were as follows: acute fracture of one-level OVCFs (T10-L2) and fractures with osteoporosis (*T*-score < −2.5), both confirmed by magnetic resonance imaging (MRI). The exclusion criteria were as follows: metastatic fractures or primary tumor or high energy trauma fractures (such as the causes by car accidents, fall from height, sport, etc.), allergy to local anesthetic drugs, and must have no history of hypertension.

The patient was placed in a prone position. PV was performed bilaterally or unilaterally under the Ziehm Imaging Systems (Ziehm Imaging GmbH, Germany) using local anesthesia. In group A, 2% of the lidocaine was diluted to 1%, in group B, 1% of the ropivacaine was diluted to 0.5%, and in group C, 1% lidocaine and 0.5% ropivacaine were mixed, for the local anesthesia. The total volume of local anesthetic drugs in each group was 20 ml. The injection technique of local anesthetic drugs is described as follows: the skin injection for a pimple, the subcutaneous tissue injection containing about 1–2 ml of anesthetic drug, the deep fascia injection containing about 5 ml of anesthetic drug, the intramuscular injection containing about 1–2 ml of anesthetic drug, and the superior facet joint containing about 5 ml of anesthetic drug. A bone needle was percutaneously inserted into the posterior one-third of the fractured vertebral body. The working cannula was transpedicularly advanced into the vertebral body. Afterward, polymethylmethacrylate (PMMA) was slowly injected into the fractured body. The surgical hemorrhage, surgical time, and cement volume were recorded accordingly.

For all the patients, the following sets of data were observed pre-operatively, post-operatively, and during the operation: (1) the surgery time, surgical hemorrhage, hospital stay, cement volume, (2) the visual analog scale (VAS), blood pressure (BP), heart rate (HR), blood oxygen saturation (BOS), surgery time, and adverse reactions. Furthermore, these data were recorded at points of before puncture, during the puncture, cement injection, and after surgery. The VAS was evaluated by two independent assessors. The 0 point indicates no pain, and the 10 point indicates the most severe pain that is unbearable.

All statistical data were analyzed with Statistic Analysis System (SAS Institute Inc., Cary, NC, USA). Quantitative data are presented as means and standard deviations. The paired-sample *t*-tests were applied for comparisons within the groups, and independent-sample *t*-tests were applied for comparisons between the groups. The correlation analysis was performed for multiple comparisons. Statistical significance was defined as a *p*-value < 0.05.

## Results

The mean age of 96 patients was 74.13 ± 7.02 years in group A, 70.47 ± 5.5 years in group B, and 73.07 ± 7.51 years in group C, without significant difference (*P* > 0.05). No significant differences were found in sex, age, hospital stay, surgery time, blood loss, and cement volume (Table 1).

**Table 1:** Characteristics and clinical findings.

**Index**	**Group A**	**Group B**	**Group C**	**P_**AB**_**	**P_**AC**_**	**P_**BC**_**
Male/Female (*n*)	11/22	10/21	12/20	0.0805	0.3126	0.4929
Mean age (years)	74.13 ± 7.02	70.47 ± 5.50	73.07 ± 7.51	0.3085	0.3466	0.6418
Hospital stay (days)	6.80 ± 1.47	6.86 ± 1.68	6.60 ± 1.24	0.9801	0.8601	0.9236
Surgery time (min)	39.67 ± 13.26	37.53 ± 9.71	40.73 ± 10.75	0.9423	0.9210	0.8650
Blood loss (ml)	7.00 ± 2.54	6.33 ± 2.29	7.33 ± 3.20	0.5314	0.7570	0.3966
Cement volume (ml)	5.02 ± 0.73	4.70 ± 0.98	4.50 ± 0.84	0.7524	0.5360	0.7551

Among the groups, during the periods of the puncture, cement injection, and immediately after surgery, the VAS in group C decreased significantly. However, the VAS showed no significant difference in the periods before surgery and 4-h after surgery among the groups (Table 2). Overall, there were no significant differences in systolic BP, diastolic BP, HR, and BOS during different periods among the groups, except HR in the period of the puncture in group C, which was slower than that in groups A and B, and HR in the period of cement injection in group A, which was faster than the other two groups (Table 3).

**Table 2:** The VAS during the different periods.

**Index**	**Group A**	**Group B**	**Group C**	**P_**AB**_**	**P_**AC**_**	**P_**BC**_**
Before surgery	4.80 ± 0.68	4.93 ± 1.10	5.00 ± 0.85	0.9729	0.7953	0.7338
Puncture	5.13 ± 0.52^*^	4.53 ± 0.74^*^	4.33 ± 0.49^*^	0.2546	0.0965	0.7438
Cement injection	4.73 ± 0.79^*^	4.13 ± 0.99^*^	3.33 ± 1.48^*^	0.3253	0.1963	0.6415
Immediate after surgery	2.60 ± 0.51^*^	2.46 ± 0.57^*^	1.53 ± 0.52^*^	0.4219	0.0347	0.0459
4-hour after surgery	1.66 ± 0.49^*^	1.46 ± 0.52^*^	0.93 ± 0.48^*^	0.4429	0.0002	0.0003

* *p < 0.05 VAS vs. before the surgery*.

**Table 3:** Systolic blood pressure (BP), diastolic BP, heart rate (HR), blood oxygen saturation (BOS) during the different periods.

**Index**	**Group A**	**Group B**	**Group C**	**P_**AB**_**	**P_**AC**_**	**P_**BC**_**
**Before surgery**						
Systolic BP	152.1 ± 13.24	148.3 ± 10.91	149.1 ± 9.67	0.4985	0.4843	0.7891
Diastolic BP	81.0 ± 5.32	85.53 ± 4.85	82.67 ± 4.67	0.4018	0.2879	0.4366
HR	96.47 ± 6.53	92.60 ± 7.53	93.20 ± 8.57	0.0591	0.0527	0.4699
BOS	98.33 ± 0.89	98.13 ± 0.92	98.33 ± 0.81	0.8454	0.8835	0.8024
**Puncture**						
Systolic BP	148.7 ± 10.12	148.30 ± 10.91	141.30 ± 6.17	0.2714	0.0523	0.0617
Diastolic BP	86.87 ± 5.49	85.53 ± 4.85	84.53 ± 3.56	0.4250	0.3705	0.4209
HR	93.80 ± 4.25	92.60 ± 4.53	87.67 ± 2.60	0.9725	0.0014	0.0008
BOS	98.40 ± 0.63	98.13 ± 0.92	98.93 ± 1.03	0.8824	0.8039	0.8061
**Cement injection**						
Systolic BP	141.7 ± 10.34	139.20 ± 8.28	142.70 ± 10.22	0.4219	0.2879	0.270
Diastolic BP	87.87 ± 5.34	81.73 ± 5.51	83.20 ± 5.27	0.0022	0.0082	0.6346
HR	90.00 ± 5.03	86.80 ± 3.44	85.47 ± 4.64	0.0001	0.0010	0.5190
BOS	98.33 ± 0.72	98.07 ± 0.96	98.73 ± 0.88	0.8929	0.8706	0.8789
**After surgery**						
Systolic BP	137.60 ± 7.57	140.00 ± 7.94	134.90 ± 6.84	0.4119	0.0750	0.250
Diastolic BP	79.73 ± 3.56	78.53 ± 3.64	78.20 ± 3.47	0.6040	0.5089	0.8563
HR	79.60 ± 3.81	79.60 ± 3.81	79.60 ± 3.84	0.9078	0.9182	0.9249
BOS	98.73 ± 0.79	98.87 ± 0.74	98.33 ± 0.72	0.8488	0.8979	0.8718

**Figure 1:**
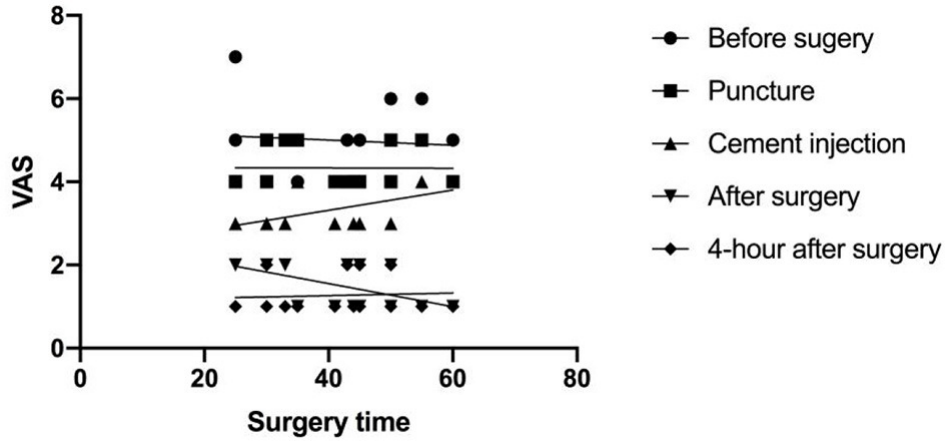
The correlation of the visual analog scale (VAS) and the surgery time.

A correlation analysis was performed between the VAS and the surgery time. In group C, a correlation was observed between the VAS and the period of cement injection (*r* = 0.5358, *p* = 0.0395), as well as between the VAS and the period of immediately after surgery (*r* = 0.5775, *p* = 0.0242). However, there was no correlation with surgery procedures in group A or in group B (Figure 1, Table 4).

**Table 4:** A correlation analysis between the visual analog scale (VAS) and the surgery time.

**Index VAS**	**The surgery time**			**rA**	**rB**	**rC**
	**Group A**	**Group B**	**Group C**			
Before surgery	0.4588	0.0599	0.7806	0.2072	−0.5048	−0.0786
Puncture	0.6930	0.2870	0.9744	0.1113	0.2943	−0.009
Cement injection	0.6548	0.2133	0.0395	0.1259	0.3412	0.5358
Immediate after surgery	0.2470	0.2468	0.0242	−0.3187	−0.3188	0.5775
4-h after surgery	0.2868	0.8692	0.7944	−0.2944	0.04654	0.0735

## Discussion

Osteoporotic vertebral compression fractures are considered as stable injuries and the patients are treated conservatively with bed rest, analgesics, brace, early rehabilitation, and osteoporosis treatments; however, they tend to progressively collapse, resulting in chronic pain, progressive kyphosis, or even delayed paralysis (1–5). Surgical managements such as PV or PK are widely preferred for vertebral height restoration, kyphosis correction, and pain relief. However, patients with OVCFs often suffer many comorbidities, and general anesthesia provides more challenges and risks (6, 9, 10). Contrarily, local anesthesia not only reduces the surgery risks but also enables patients to recover quickly after the operation. However, local anesthesia still faces problems such as poor effect and poor patient experience. Although the surgeons use analgesics to reduce patients' intraoperative pain, adverse reactions could bring more risks for geriatric patients. How to improve the effectiveness of local anesthesia still remains an issue (11–17).

During PV, our team used lidocaine as a routine local anesthetic that works fast but does not last too long. Hence, the patients always suffered or the local anesthesia was changed into general anesthesia. Therefore, finding a suitable method for relieving the pain is key to PV surgery. Ropivacaine works in about 15 min and could last 3–5 h. In our study, groups B and C could obtain the affirmative effect of local anesthesia, and the VAS was observed to decrease significantly. Especially in group C, the VAS decreased significantly during the periods of the puncture, cement injection, and immediately after surger. Furthermore, a correlation was observed between the VAS and the periods of cement injection and after surgery. These factors could ensure the cooperation of patients in the surgery and also help maintain enough anesthesia time for the surgeon, ensuring patients' safety.

Furthermore, there were no significant differences in systolic BP, diastolic BP, HR, and BOS during different periods among the groups, except HR in the period of the puncture in group C, which was slower than that in group A and B, and HR in the period of cement injection in the group A, which was faster than the other two groups. We noticed that the application combination of lidocaine and ropivacaine has a fast and lasting effect, stable intraoperative circulation and breathing, small physiological disturbances, and short post-operative recovery time. Hence, the combination of short-acting local anesthetic and long-acting local anesthetic could satisfy the surgery, reduce surgical complications, and improve the quality of surgery which could provide a new way in the treatment of OVCFs with PV.

When multiple levels of OVCFs need PV treatment or the patients could not tolerate the pain, the patients prefer general anesthesia. Although general anesthesia makes the patients more comfortable, surgeons and patients face more risks without intraoperative neurophysiological monitoring. With the increase in the geriatric population, older adults have many comorbidities, and the risk of general anesthesia is high (18–22). De Berti et al. (23) introduced a method of the administration of conscious sedation by a neuroradiology team for PV and spinal biopsy procedures, which could observe that conscious sedation can be safely administered. However, this method needs a specialized and well-trained team and angiography equipment. Liu et al. (24) suggested extrapedicular infiltration anesthesia as an improved method of local anesthesia for unipedicular PV or PK and no adverse nerve root effects or complications were recorded. Zhang et al. (25) reported that they performed the vertebral cancellous bone infiltration anesthesia may effectively relieve intraoperative pain and improve the surgical experience of patients. However, lidocaine has a problem of resulting in serious adverse reactions if injected too quickly, with a risk of entering the venous system rapidly.

Our study has a few limitations. First, the retrospective nature of the study indicates the possibility of bias. Second, the study was limited to two local anesthetic drugs because there are more management for relieving the pain during the surgery, such as analgesics, opioids drugs, and sedatives. Third, patients with OVCFs treated with PK were not included, and only one-level OVCFs were discussed. Fourth, the VAS was assessed during the puncture and cement injection, which may not be accurate and may be related to bias. Fifth, the study was focused on the applications of local anesthesia in PV, but other clinical outcomes were not observed. In the future, prospective, randomized, and grouped studies with long-term follow-up periods are needed.

## Conclusion

For the first time, the present study investigated the three application methods of local anesthetics in PV, which were reliable and safe. Compared with the other two methods, the use of lidocaine in combination with ropivacaine could effectively relieve intraoperative pain, making the patients more comfortable and experience better recovery.

## Data Availability Statement

The raw data supporting the conclusions of this article will be made available by the authors, without undue reservation.

## Ethics Statement

The studies involving human participants were reviewed and approved by the Institutional Review Board of the First Affiliated Hospital of Chongqing Medical University. The patients/participants provided their written informed consent to participate in this study.

## Author Contributions

WZ and YO contributed to the study design. WZ performed the surgery. JX and YO collected and analyzed the data. JX and WZ wrote the manuscript. All authors read and approved the final manuscript.

## Funding

This study was supported by the Hospital Training Funding (PYJJ2019-08) and the Medical Program of Chongqing Health and Science, Technology Commission (2021MSXM285). The funding body has not been involved in the design, data collection, analysis, interpretation, or writing of the manuscript.

## Conflict of Interest

The authors declare that the research was conducted in the absence of any commercial or financial relationships that could be construed as a potential conflict of interest.

## Publisher's Note

All claims expressed in this article are solely those of the authors and do not necessarily represent those of their affiliated organizations, or those of the publisher, the editors and the reviewers. Any product that may be evaluated in this article, or claim that may be made by its manufacturer, is not guaranteed or endorsed by the publisher.
